# East Tennessee Dental Association

**Published:** 1869-12

**Authors:** W. F. Fowler

**Affiliations:** Greeneville, Tenn.


### ARTICLE V.

### *East Tennessee Dental Association.*

By Dr. W. F. Fowler, Greeneville, Tenn.

The East Tenn. Dental Association met pursuant to adjournment, at the Lamar House, Knoxville, Tenn., October 20th 1869, at 3 P. M.

The Association was called to order by the President Dr. John Fouche, who ordered the minutes of the last meeting to be read, after which several new members were elected. The increase in members, both upon the rolls and of members present, show the growing interest manifested in this body and its objects. An interesting paper on Hæmorrhage after Extraction was read by Dr. Wm. F. Fowler—the various remedies and means for arresting the same were discussed ; after which the Association adjourned to meet next morning at 8 o'clock.

SECOND DAY.

The morning was occupied by a very interesting and instructive clinic by Dr. J. T. Cazier.

The rest of the day was consumed by discussions upon the various methods of treating Superficial Decay, removing Salivary Calculus &c., also the different methods of filling teeth.

THIRD DAY.

A clinic also was held by Dr. John Fouche in the forenoon, which was witnessed also with the greatest of interest, the Association feeling greatly benefitted by the clinics of the two mornings. Two Essays were read by Dr. Wm. H. Cooke, one on Necrosis of the Alveoli, the other on Tumors of the Mouth; both were highly entertaining and instructive. Dr. Cazier drew up and presented to the Association a bill memorializing the Legislature of the State of Tennessee to pass the same, for the better regulating the practice of Dentistry in Tennesssee.

Drs. Fouche, Cazier and Cooke were elected to represent the Association at the next meeting of the American Dental Association, which meets in the city of Nashville. Also Drs. Fowler and Speck were elected to represent the interests of the same, at the next meeting of the Southern Dental Association at New Orleans. Several members having to leave, the Association adjourned after electing the following officers for the ensuing year, to wit:

*President.*—J. T. CAZIER, D.D.S.
*Cor. Secretary.*—WM. F. FOWLER, D.D.S.
*Rec. Sec. and Treas.*—WM. H. COOKE.

---

